# Factors of the reproductive life plan for married and employed Japanese women considering having children

**DOI:** 10.1186/s12905-026-04600-1

**Published:** 2026-06-13

**Authors:** Shizuko Angerhofer, Mikako Yoshida, Fumi Atogami, Yasuka Nakamura, Yoko Takeishi, Toyoko Yoshizawa

**Affiliations:** 1 Faculty of Nursing, Iwate Prefectural University, 152-52, Sugo, Takizawa, Iwate 020-0693 Japan; 2 Department of Women’s Health & Midwifery, Tohoku University Graduate School of Medicine, Sendai, Miyagi Japan; 3 Faculty of Life Sciences, Kumamoto University, Kumamoto, Kumamoto Japan; 4 Department of Nursing, Yamagata Prefectural University of Health Sciences, Yamagata, Yamagata Japan; 5 Mie Prefectural College of Nursing, Tsu, Mie Japan

**Keywords:** Career vision, Fertility, Knowledge, Pregnancy, Reproductive behavior, Reproductive vision, Theory of planned behavior

## Abstract

**Background:**

Women’s delayed childbearing results in negative effects on maternal health and may restrict women from achieving their reproductive goals. By the age of 30 years, Japanese women are typically in the process of acquiring a more autonomous and fulfilling work status. Conversely, this is also the time when women experience a decline in fertility. Therefore, it is necessary to explore how reproductive and career visions affect intentions and behaviors toward the Reproductive Life Plan (RLP) to build support for achieving their reproductive goals. The objective of this study was to examine the associations between RLP and the reproductive and career visions of employed, married, and childless Japanese women in their 30s.

**Methods:**

We administered a questionnaire comprising 29 items on RLP (i.e., pregnancy timing considerations and attitudes favoring pregnancy delay), reproductive and career visions, and participants’ demographics. A multi-logistic regression analysis was used to explore the factors influencing “absence of pregnancy timing consideration” and “attitude favoring pregnancy delay.”

**Results:**

Approximately half of the participants had completed a university education, most of them were in non-management positions, and 36.1% were working 40 h or more per week. The average number of desired children was 1.7 ± 0.6. The factor “absence of pregnancy timing consideration” was associated with education, marriage duration, infertility concerns, self-perceived pregnancy probability, the number of desired children, and the intention to return to work after delivery. “Attitude favoring pregnancy delay” was associated with infertility concerns, scores of fertility knowledge, the interval between the ideal age for the first pregnancy and current age, the number of desired children, and working more than 40 h per week.

**Conclusion:**

Absence of pregnancy timing consideration” and “Attitude favoring pregnancy delay” were associated with different factors. Developing support systems for RLP, including the development of reproductive and career visions, will help prevent delayed childbearing among employed, married, and childless Japanese women in their 30s.

**Supplementary Information:**

The online version contains supplementary material available at https://doi.org/10.1186/s12905-026-04600-1.

## Background

The total fertility rate in Japan declined to 1.15 in 2024, reaching its lowest level on record [1]. Further, the average age at first birth has risen steadily and now exceeds 30 years, placing Japan among the highest of all high-income countries [2]. In Japan, 65% of women aged 30–39 are married [3]. Although most married women in their 30 s want children, a significant number remains childless, suggesting persistent difficulties in achieving their reproductive goals. Given the well-established age-related decline in fertility, delayed childbearing has become a pressing concern, as it increases the likelihood of infertility, pregnancy complications such as gestational diabetes and hypertensive disorders, and adverse birth outcomes including preterm birth [4–9].

Women’s considerations and actions regarding the timing of pregnancy, apart from the decision to delay pregnancy, are aspects of what is called the *Reproductive Life Plan* (RLP), which includes both decision-making and collaborative processes between women and their healthcare providers [10]. The RLP refers to one’s life planning regarding all reproductive aspects, such as considerations involved in determining whether or not to have children or the ideal timing of childbearing among women who plan to have children in the future.

One of the factors that affect RLP is *reproductive vision*, which is based on one’s reproductive health knowledge (e.g., knowledge about one’s fertility stages and periods, also known as *fertility knowledge*). Fertility knowledge refers to one’s understanding of pregnancy mechanisms, sexually transmitted infections (STIs), pregnancy capabilities, and the factors influencing pregnancy capability, such as age-related fertility decline and adverse pregnancy outcomes due to smoking [11, 12]. In Japan, one of the reasons behind delayed childbearing may be low fertility knowledge. Reportedly, sexuality education provided to Japanese adolescents focuses on preventing unintended pregnancy and STIs, and does not sufficiently address fertility knowledge [13, 14]. Moreover, fear of unintended pregnancy could fuel the misinformation that women can get pregnant at any time. Furthermore, previous studies have reported that the media are a primary source of fertility knowledge for Japanese women [12, 15], and its portrayals frequently highlight pregnancies among celebrities in their 40 s, which may contribute to misperceptions about natural fertility potential at advanced maternal ages.

Another factor affecting RLP is *career vision*, which is essential for Japanese women in their 30s. Historically, in Japanese culture, women were expected to focus on housework after marriage or childbirth, regardless of their views on the subject [16]. Furthermore, Japanese culture has yet to fully develop a clear understanding of the need and possibility for women to have careers after they get married or become pregnant. In professional lives, Japanese individuals tend to reach a working status that denotes greater autonomy and work fulfillment around their 30s. Additionally, this specific period is particularly important for career development considerations [17]. Therefore, women in this age group find themselves in difficult situations, as they need to pursue future career development while trying to align potential career growth with personal decisions, such as whether or not to have children, the reality that having children involves maternity leave, and the need to appropriately plan and manage their actions if they intend to have healthy pregnancies in the near or distant future.

Previous studies reported that enhanced reproductive and career visions were associated with the intention to consider the timing of pregnancy [11, 18–21]. Additionally, two studies focusing on behaviors related to delayed pregnancy timing found that women who had fertility knowledge and perceived support from their workplace in case they became pregnant were more likely to attempt pregnancy [22, 23]. However, these studies typically addressed one dimension of RLP at a time, and evidence remains limited on how reproductive and career factors jointly influence both pregnancy timing consideration (intention) and attitudes favoring pregnancy delay (behavior) [24–27]. Since intent does not always lead to actual behavior [28], it is essential to examine these two aspects simultaneously to better understand the mechanisms contributing to delayed childbearing.

Therefore, we aimed to examine which factors of reproductive and career vision are associated with RLP (including intention, namely, “*pregnancy timing consideration,*” and behavior, namely, “*attitude favoring pregnancy delay”*) among employed, married, and childless Japanese women in their 30s. Understanding the determinants of both intention and behavior is necessary for informing future policy and educational interventions aimed at supporting timely childbearing and addressing the rising trend of delayed pregnancy in Japan.

## Methods

### Design

This study used an online cross-sectional survey design and was conducted between June 2017 and July 2017.

### Participants and procedures

Women living in Japan who were employed, aged 30–39 years, and registered with a web research company (Cross Marketing Inc. Japan) were recruited by email. Inclusion criteria included being married, having no children, and wishing to have children in the future. Exclusion criteria included having undergone a hysterectomy or being unable to understand Japanese. Participants enrolled in the study accessed the questionnaire website voluntarily and answered screening items pertaining to the inclusion and exclusion criteria (e.g., number of children, marital status, and desire to have children in the future). Those who met the inclusion criteria proceeded to the agreement page and, after agreeing to the terms, accessed the survey.

### Measures

**Fig. 1:**
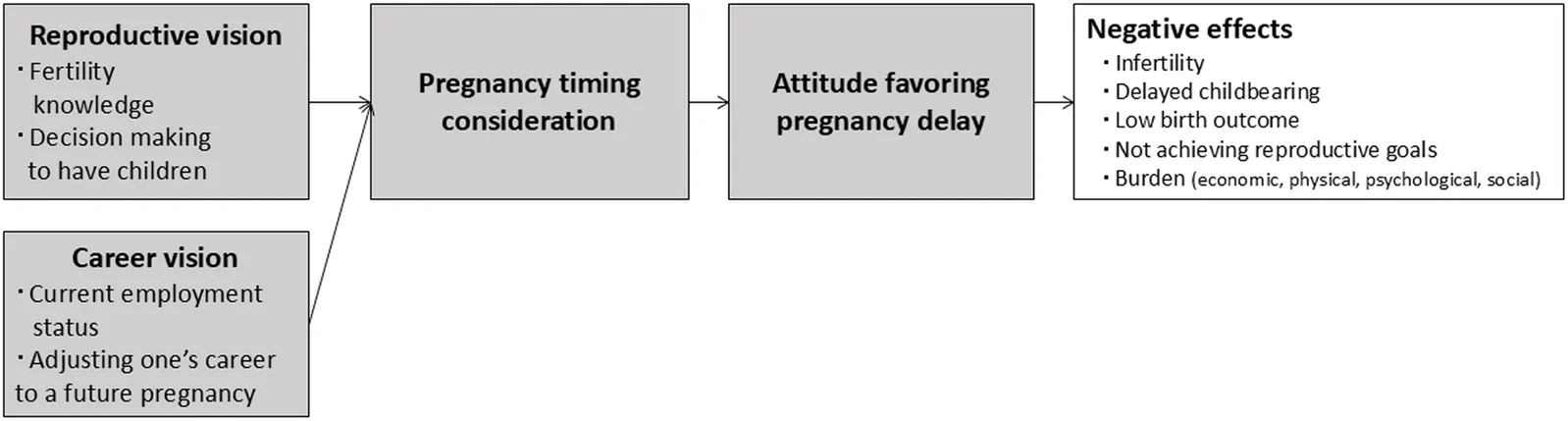
Conceptual framework

The questionnaire developed by the authors [29] included participants’ RLP, which encompassed pregnancy timing considerations, attitudes favoring pregnancy delay, reproductive and career vision, and demographic characteristics (Fig. 1). In this study, we defined reproductive vision as one’s reproductive health knowledge and decision-making regarding having children, and career vision as one’s current employment status and plans for adjusting one’s career in relation to pregnancy. To achieve the study's aims, reproductive and career visions were established as independent variables.

Figure 1 illustrates the conceptual diagram of this study; the arrows in the figure indicate the influence that these two visions have on delayed childbearing, which comprises "Pregnancy timing consideration" and "Attitude favoring pregnancy delay" among the participants. According to the Theory of Planned Behavior [28], people’s intentions are the immediate antecedents of their corresponding behavior; further, intentions are assumed to capture the motivational factors that influence a given behavior. Since intention and behavior are not conceptually equal, we believe they warrant separate examinations. Based on this theory, for the purpose of this study, "Pregnancy timing consideration" was analyzed for pregnancy intention, and "Attitude favoring pregnancy delay" was analyzed for pregnancy behavior.

#### RLP

##### Pregnancy timing consideration and attitude favoring pregnancy delay

Both pregnancy timing consideration and attitude favoring pregnancy delay were evaluated using the same question: “Would you be okay if you were to get pregnant right now?” Participants had four possible responses to this question: “Right now would be okay,” “Not now owing to work-related reasons,” “Not now owing to non-work-related reasons,” and “Never considered.” When a participant answered “Never considered,” the response was categorized as “*absence of pregnancy timing consideration*,” and when a participant selected either of the two middle responses (“Not now owing to […]”), it was categorized as “*attitude favoring pregnancy delay*.”

##### Reproductive vision. Fertility knowledge

Fertility knowledge was assessed using 13 questions developed based on the Cardiff Fertility Knowledge Scale [11], a literature review [30–33], and data on fertility in Japan [34]. These questions were categorized as “True,” “False,” and “Don’t know.” A correct answer received 1 point, while incorrect or “Don’t know” responses received 0 points. The points were then summed, divided by 13, and multiplied by 100, producing a percentage indicating the accuracy of women's fertility knowledge. The resulting score ranged from 0–100%, with a higher percentage indicating more thorough and accurate fertility knowledge. The internal reliability of the fertility knowledge questionnaire was high, with a standardized Cronbach α coefficient of 0.86.

The question measuring *self-perceived pregnancy probability* was “What would be the chance, in percentage, for you to get pregnant?” The question measuring *infertility concerns* was “Have you ever been concerned about having infertility issues?” S*elf-perceived possible pregnancy age* was defined as the self-perceived maximum age at which one could still get pregnant, and the question measuring it was “What would be the maximum age that you deem would still allow for you to naturally have children?” The response to this question was subtracted from the women’s current age at the time of the research.

##### Decision making to have children

First, we asked participants about the number of desired children. Then, we used a question to measure *self-perceived ideal age for the first and last pregnancy*, which was, “At what age do you want to have your first/last child?” The response to this question was used as the *ideal pregnancy age*; that is, this concept was measured through women’s responses regarding which age they deemed ideal for them to get pregnant for the first and last time. To clarify the interval between women’s current age (at the time of research) and their ideal first and last pregnancy ages, these two were subtracted from the current age.

##### Career vision

Career vision included questions about participants’ *work conditions* and their *perceptions* regarding *returning to work after delivery*. To measure work conditions, we used the following items: *employment type* (3 categories: non-regular status with no maternity leave; non-regular status with maternity leave; regular status), *position* (2 categories: non-management; management), *working hours per week* (2 categories: less than 40; 40 or more), and *support from co-workers* (4 categories: none; not much; some; very much). Maternity leave in this study encompassed both a 6- to 8-week postpartum leave and a 1- to 3-year leave after childbirth. Although legally recognized rights exist, awareness of maternity and childcare leave among non-regular employees remains low [35]. Therefore, this survey asked participants to respond based on their own understanding of their status. The question regarding participants’ perceptions of returning to work after delivery was, “How would your return to work after delivery be?” Responses were categorized into three options: “quit”, “not sure” and “return.”

#### Demographic characteristics

Regarding participants’ demographic characteristics, questions focused on their age, education, husbands’ age, and marriage duration.

### Data analysis

A 2-sample proportion comparison, with 80% power and at the 5% level of significance, required a sample of 64 women to measure pregnancy timing considerations. According to a previous study [20], 15% of women in their sample cohort had never considered pregnancy; hence, we deduced a final sample size of 500 for our study.

To explore the factors influencing the *absence of pregnancy timing consideration*, participants were categorized into two groups based on four answers regarding pregnancy timing consideration: 1) Never considered (those who chose this answer regarding their RLP question, and the other groups followed this pattern), or 2) Right now, Not now because of work-related reasons, and Not now owing to non-work-related reasons. To explore the factors influencing the *attitude favoring pregnancy delay*, participants were categorized into two groups based on three answers: 1) the Favoring pregnancy delay attitude group (comprising participants who answered either "Not now" owing to work-related reasons or "Not now" owing to non-work-related reasons regarding their RLP question), and 2) the Right now group (comprising those who answered "Right now would be okay"). Those in the “Right now” group were considered to have an attitude attempting pregnancy. Spearman’s correlation coefficient or chi-square tests were used to assess multicollinearity.

We used one-way analysis of variance (ANOVA) and chi-square tests for continuous and categorical variables, respectively, to identify differences in reproductive and career vision among each pregnancy timing consideration group. A multi-logistic regression analysis was performed to estimate the odds ratio (OR) and 95% confidence interval (CI) of reproductive and career vision toward participants’ RLP. Statistical analysis was performed using IBM SPSS for Windows ver. 25 (IBM Corp., Armonk, NY), and the level of statistical significance was set at a *p* < 0.05.

## Results

In total, 498 women participated in this study. Nonetheless, two participants who marked the same choices for all questions were excluded, resulting in 496 eligible participants whose data were analyzed. Participants’ average age was 34.5 ± 2.8 years; about half (48.4%) had completed undergraduate or graduate education; husbands’ average age was 36.4 ± 5.4 years; average marriage duration was 4.4 ± 3.1 years, with 12 (2.4%) having been married for less than 1 year and 36 (7.3%) having been married for 10 years or more.

For pregnancy timing, 354 participants (71.4%) answered “Right now would be okay,” while 37 (7.5%) answered “Never considered.” Compared with the other three groups, the Never considered group showed significant differences in age (both the wife’s and husband’s age) and marriage duration (p ≤ 0.01, Table 1). Regarding self-perceived pregnancy probability, the Never considered group showed a significant difference compared with the “Not now” groups (*p* < 0.01, Table 1). Moreover, compared with the “Not now owing to work-related reasons” group and the “Never considered” group, the “Right now” group showed significant differences in the score of fertility knowledge (*p* < 0.001, Table 1). Additionally, compared with the “Not now owing to non-work-related reasons” group and the “Never considered” group, the “Right now” group showed significant differences in the number of desired children (*p* < 0.001, Table 1). When compared with the two “Not now” groups, the “Right now” group showed significantly lower values for the intervals between possible pregnancy age and current age, ideal age for first pregnancy and current age, and ideal age for last pregnancy and current age (*p* < 0.01, Table 1).

**Table 1:** Participants’ demographic, reproductive vision, and career vision characteristics compared by pregnancy timing answers (*n* = 496)

	A) Right now would be okay (*n* = 354)	B) Not now owing to work-related reasons (*n* = 46)	C) Not now owing to non-work-related reasons (*n* = 59)	D) Never considered this (*n* = 37)	*p* value	Multiple comparison
Wife’s age (years)	34.6 ± 2.7	33.1 ± 2.6	33.9 ± 2.6	35.9 ± 2.6	< 0.001	B < A** A < D* B < D*** C < D**
30–34	164 (46.3%)	34 (73.9%)	38 (64.4%)	11 (29.7%)	< 0.001	
35–39	190 (53.7%)	12 (26.1%)	21 (35.6%)	26 (70.3%)		
Education						
Junior college or less	177 (50.0%)	24 (52.2%)	28 (47.5%)	27 (73.0%)	0.056	
Undergraduate or graduate	177 (50.0%)	22 (47.8%)	31 (52.5%)	10 (27.0%)		
Husband’s age (years)	36.3 ± 5.1	35.2 ± 5.3	35.9 ± 6.0	40.0 ± 6.1	0.001	A < D** B < D** C < D**
≤ 35	153 (44.4%)	28 (60.9%)	34 (57.6%)	11 (29.7%)	0.009	
≥ 36	197 (55.6%)	18 (39.1%)	25 (42.4%)	26 (70.3%)		
Marriage duration (years)	4.4 ± 3.0	3.4 ± 2.6	3.5 ± 3.0	7.0 ± 3.8	< 0.001	A < D*** B < D*** C < D***
≤ 3	161 (45.5%)	30 (65.2%)	36 (61.0%)	8 (21.6%)	< 0.001	
≥ 4	193 (54.5%)	16 (34.8%)	23 (39.0%)	29 (78.4%)		
Reproductive Vision						
Fertility knowledge score (points)	58.8 ± 25.3	45.8 ± 24.9	51.1 ± 24.6	38.9 ± 29.3	< 0.001	B < A** D < A***
≤ 54	152 (42.9%)	30 (65.2%)	39 (66.1%)	27 (73.0%)	< 0.001	
≥ 55	202 (57.1%)	16 (34.8%)	20 (33.9%)	10 (27.0%)		
Self-perceived pregnancy probability (%)	38.9 ± 26.6	52.8 ± 22.0	45.6 ± 26.8	32.3 ± 21.5	< 0.001	A < B** D < B*** D < C*
≤ 49	184 (52.0%)	11 (23.9%)	22 (37.3%)	20 (54.1%)	< 0.001	
50	100 (28.2%)	18 (39.1%)	18 (30.5%)	15 (40.5%)		
≥ 51	70 (19.8%)	17 (37.0%)	19 (32.2%)	2 (5.4%)		
Infertility concerns						
No	34 (9.6%)	14 (30.4%)	21 (35.6%)	17 (45.9%)	< 0.001	
Yes	320 (90.4%)	32 69.6%)	38 (64.4%)	20 (54.1%)		
Interval between possible pregnancy age and current age (years)	4.5 ± 2.9	6.1 ± 3.3	6.4 ± 3.8	4.7 ± 3.5	< 0.001	A < B** A < C***
≤ 4	187 (52.8%)	15 (32.6%)	14 (23.7%)	20 (54.1%)	< 0.001	
≥ 5	167 (47.2%)	31 (67.4%)	45 (76.3%)	17 (45.9%)		
Interval between the ideal age for first pregnancy and current age (years)	0.5 ± 2.6	2.2 ± 1.3	2.0 ± 1.4	1.1 ± 2.0	< 0.001	A < B*** A < C***
≤ 1	287 (81.1%)	16 (34.8%)	19 (32.2%)	23 (62.2%)	< 0.001	
≥ 2	67 (18.9%)	30 (65.2%)	40 (67.8%)	14 (37.8%)		
Interval between the ideal age for last pregnancy and current age (years)	3.2 ± 2.1	4.5 ± 2.0	3.7 ± 1.8	3.1 ± 2.4	0.001	A < B** D < B**
≤ 3	201 (56.8%)	12 (26.1%)	27 (45.8%)	21 (56.8%)	0.001	
≥ 4	153 (43.2%)	34 (73.9%)	32 (54.2%)	16 (43.2%)		
Number of desired children	1.8 ± 0.6	1.7 ± 0.7	1.5 ± 0.6	1.4 ± 0.6	< 0.001	C < A** D < A**
1	102 (28.8%)	19 (41.3%)	33 (55.9%)	24 (64.9%)	< 0.001	
≥ 2	252 (71.2%)	27 (58.7%)	26 (44.1%)	13 (35.1%)		
Career Vision						
Employment type						
Non-regular: No maternity leave	150 (42.4%)	19 (41.3%)	23 (39.0%)	17 (46.0%)	0.170	
Non-regular: Maternity leave	45 (12.7%)	3 (6.5%)	13 (22.0%)	8 (21.6%)		
Regular	159 (44.9%)	24 (52.2%)	23 (39.0%)	12 (32.4%)		
Position						
Non-management	341 (96.3%)	42 (91.3%)	59 (100%)	34 (91.9%)	0.080	
Management	12 (3.7%)	4 (8.7%)	0 (0%)	3 (8.1%)		
Working hours per week						
< 40	232 (65.5%)	22 (47.8%)	36 (61.0%)	27 (73.0%)	0.069	
≥ 40	122 (34.5%)	24 (52.2%)	23 (39.0%)	10 (27.0%)		
Support from co-workers						
None	22 (6.2%)	4 (8.7%)	2 (3.4%)	5 (13.5%)	0.043	
Not much	49 (13.8%)	20 (43.5%)	7 (11.9%)	5 (13.5%)		
Some	162 (45.8%)	13 (28.3%)	25 (42.4%)	20 (54.1%)		
Very much	121 (34.2%)	9 (19.6%)	25 (42.4%)	7 (18.9%)		
Return to work after delivery						
Quit	50 (14.1%)	7 (15.2%)	9 (15.3%)	11 (29.7%)	0.033	
Not sure	114 (32.2%)	9 (19.6%)	14 (23.7%)	14 (37.8%)		
Return	190 (53.7%)	30 (65.2%)	36 (61.0%)	12 (32.4%)		

Data are presented as mean ± SD or n (%). Statistical analyses were performed using one-way ANOVA with multiple comparison tests (Tukey, Games-Howell); and chi-square tests

^*^p < 0.05, **p < 0.01, ***p < 0.001

Regarding the absence of pregnancy timing consideration (defined as responding “Never considered” to the RLP question; Table 2), it was significantly associated with higher education (adjusted odds ratio [AOR] 0.38 [CI: 0.15–0.94], *p* = 0.037), longer marriage duration (AOR 2.96 [CI: 1.13–7.77], *p* = 0.028), presence of infertility concerns (AOR 0.14 [CI: 0.06–0.35], *p* < 0.001), higher self-perceived pregnancy probability (AOR 0.14 [CI: 0.03–0.76], *p* = 0.022), higher number of desired children (AOR 0.24 [CI: 0.09–0.67], *p* = 0.006), and consideration of returning to work after delivery (AOR 0.22 [CI: 0.07–0.68], *p* = 0.009). When self-perceived pregnancy probability was treated as a continuous variable, the association remained statistically significant (AOR = 0.98, *p* = 0.020).

**Table 2:** Factors associated with reproductive and career vision that influenced absence of pregnancy timing consideration (*n* = 496)

	COR	(95% CI^a^)	*p* value	AOR	(95% CI^a^)	*p* value
Wife’s age (years)						
30–34	ref			ref		
35–39	2.50	(1.02–5.18)	0.014	1.81	(0.64–5.09)	0.261
Education						
Junior college or less	ref			ref		
Undergraduate or graduate	0.37	(0.17–0.78)	0.009	0.38	(0.15–0.94)	0.037
Husband’s age (years)						
≤ 35	ref			ref		
≥ 36	2.16	(1.04–4.47)	0.039	1.41	(0.52–3.82)	0.500
Marriage duration (years)						
≤ 3	ref			ref		
≥ 4	3.55	(1.59–7.92)	0.002	2.96	(1.13–7.77)	0.028
Reproductive vision						
Fertility knowledge score (points)						
≤ 54	ref			ref		
≥ 55	0.34	(0.16–0.73)	0.005	0.49	(0.20–1.18)	0.113
Self-perceived pregnancy probability (%)						
≤ 49	ref			ref		
50	1.20	(0.59–2.42)	0.617	1.24	(0.52–2.10)	0.627
≥ 51	0.21	(0.05–0.89)	0.035	0.14	(0.03–0.76)	0.022
Infertility concerns						
No	ref			ref		
Yes	0.21	(0.10–0.42)	< 0.001	0.14	(0.06–0.35)	< 0.001
Interval between possible pregnancy age and current age (years)						
≤ 4	ref			ref		
≥ 5	0.76	(0.39–1.48)	0.414	1.62	(0.63–4.18)	0.320
Interval between the ideal age for first pregnancy and current age (years)						
≤ 1	ref			ref		
≥ 2	1.43	(0.72–2.86)	0.312	1.00	(0.40–2.52)	0.993
Interval between the ideal age for last pregnancy and current age (years)						
≤ 3	ref			ref		
≥ 4	0.84	(0.43–1.64)	0.601	2.66	(0.93–7.63)	0.068
Number of desired children						
1	ref			ref		
≥ 2	0.27	(0.14–0.55)	< 0.001	0.24	(0.09–0.67)	0.006
Career vision						
Employment type						
Non-regular: No maternity leave	ref			ref		
Non-regular: Maternity leave	1.48	(0.61–3.60)	0.386	2.96	(0.95–9.23)	0.061
Regular	0.66	(0.31–1.41)	0.283	0.95	(0.34–2.63)	0.915
Position						
Non-management	ref			ref		
Management	2.29	(0.64–8.22)	0.202	2.96	(0.51–17.18)	0.227
Working hours per week						
< 40	ref			ref		
≥ 40	0.64	(0.30–1.35)	0.236	1.14	(0.41–3.20)	0.798
Support from co-workers						
None	ref			ref		
Not much	0.41	(0.11–1.51)	0.179	0.82	(0.16–4.26)	0.814
Some	0.54	(0.19–1.56)	0.254	1.23	(0.29–5.16)	0.775
Very much	0.25	(0.08–0.85)	0.027	0.56	(0.11–2.91)	0.491
Return to work after delivery						
Quit	ref			ref		
Not sure	0.61	(0.26–1.42)	0.255	0.59	(0.20–1.73)	0.334
Return	0.28	(0.12–0.67)	0.004	0.22	(0.07–0.68)	0.009

Dependent Variables: 1 = Never considered, 0 = Right now would be okay, not now owing to work reasons, and not now owing to non-work reasons

^a^CI = confidence interval, Model χ^2^ (21) = 79.5, *p* < 0.001; Nagelkerke R^2^ = 0.36; Hosmer–Lemeshow test, *p* < 0.001

The attitude favoring pregnancy delay (defined as responding “Not now owing to […]” possibilities to the RLP question; Table 3) was significantly associated with the presence of infertility concerns (AOR 0.32[CI:0.16–0.63], *p* = 0.001), a higher score in fertility knowledge (AOR 0.53[CI:0.30–0.94], *p* = 0.029), a longer interval between the ideal age for the first pregnancy and current age (AOR 4.88[CI:2.72–8.75], *p* < 0.001), a higher number of desired children (AOR 0.29[CI:0.15–0.56], *p* < 0.001), and working ≥ 40 h per week (AOR 1.91[CI:1.00–3.63], *p* = 0.049).

**Table 3:** Factors associated with reproductive and career vision that influenced attitude favoring pregnancy delay (*n* = 496)

	COR	(95% CI^a^)	*p* value	AOR	(95% CI^a^)	*p* value
Wife’s age (years)						
30–34	ref			ref		
35–39	0.40	(0.25–0.63)	< 0.001	0.74	(0.37–1.44)	0.368
Education						
Junior college or less	ref			ref		
Undergraduate or graduate	1.02	(0.66–1.58)	0.093	0.90	(0.51–1.57)	0.700
Husband’s age (years)						
≤ 35	ref			ref		
≥ 36	0.55	(0.36–0.86)	0.009	0.72	(0.38–1.36)	0.307
Marriage duration (years)						
≤ 3	ref			ref		
≥ 4	0.49	(0.32–0.77)	0.002	0.75	(0.42–1.34)	0.336
Reproductive vision						
Fertility knowledge score (points)						
≤ 54	ref			ref		
≥ 55	0.39	(0.25–0.62)	< 0.001	0.53	(0.30–0.94)	0.029
Self-perceived pregnancy probability (%)						
≤ 49	ref			ref		
50	2.01	(1.18–3.42)	0.010	1.44	(0.73–2.86)	0.291
≥ 51	2.87	(1.66–4.95)	< 0.001	1.66	(0.79–3.48)	0.183
Infertility concerns						
No	ref			ref		
Yes	0.21	(0.12–0.36)	< 0.001	0.32	(0.16–0.63)	0.001
Interval between possible pregnancy age and current age (years)						
≤ 4	ref			ref		
≥ 5	2.94	(1.82–4.72)	< 0.001	1.59	(0.82–3.09)	0.170
Interval between the ideal age for first pregnancy and current age (years)						
≤ 1	ref			ref		
≥ 2	8.57	(5.27–13.92)	< 0.001	4.88	(2.72–8.75)	< 0.001
Interval between the ideal age for last pregnancy and current age (years)						
≤ 3	ref			ref		
≥ 4	2.22	(1.42–3.48)	< 0.001	1.63	(0.80–3.31)	0.178
Number of desired children						
1	ref			ref		
≥ 2	0.41	(0.26–0.65)	< 0.001	0.29	(0.15–0.56)	< 0.001
Career vision						
Employment type						
Non-regular: No maternity leave	ref			ref		
Non-regular: Maternity leave	1.27	(0.65–2.47)	0.482	1.11	(0.47–2.61)	0.812
Regular	1.06	(0.66–1.69)	0.822	0.71	(0.35–1.43)	0.338
Position						
Non-management	ref			ref		
Management	1.04	(0.33–3.26)	0.948	1.01	(0.26–3.92)	0.994
Working hours per week						
< 40	ref			ref		
≥ 40	1.54	(0.99–2.40)	0.056	1.91	(1.00–3.63)	0.049
Support from co-workers						
None	ref			ref		
Not much	1.50	(0.53–4.24)	0.448	1.33	(0.36–5.00)	0.670
Some	1.02	(0.39–2.66)	0.970	0.80	(0.23–2.77)	0.729
Very much	1.03	(0.39–2.74)	0.952	0.98	(0.28–3.48)	0.975
Return to Work After Delivery						
Quit	ref			ref		
Not sure	0.63	(0.31–1.30)	0.209	0.54	(0.22–1.33)	0.180
Return	1.09	(0.58–2.04)	0.798	0.99	(0.42–2.35)	0.915

Dependent Variables: 1 = Not now owing to work reasons, and not now owing to non-work reasons, 0 = Right now would be okay

^a^CI = confidence interval, Model χ^2^ (21) = 145.8, *p* < 0.001; Nagelkerke R^2^ = 0.41; Hosmer–Lemeshow test, *p* = 0.35

Multicollinearity was assessed using Spearman’s correlation coefficients and chi-square tests, and no variable pairs showed problematic correlations.

## Discussion

This study revealed that reproductive vision and career vision were associated with the ‘*absence of pregnancy timing consideration*’ and ‘*attitude favoring pregnancy delay*’. Furthermore, infertility concerns related to reproductive vision were common in the context of ‘absence of pregnancy timing consideration’ and ‘attitude favoring pregnancy delay’, while some related factors of reproductive vision and career vision differed in these concepts; the idea of returning to work after delivery decreased the risk of ‘absence of pregnancy timing consideration’, and fertility knowledge and working shorter hours prevented the ‘attitude favoring pregnancy delay’.

Our results showed that having a higher level of education, higher self-perceived pregnancy probability, greater number of desired children, and presence of infertility concerns and thoughts about returning to work after delivery were associated with lower likelihood of opting for absence of pregnancy timing consideration. Our finding regarding the relationship between infertility concerns and the absence of pregnancy timing consideration is consistent with that reported previously; McQuillan [22] reported that women trying to become pregnant were more likely to self-identify as infertile because the presence of infertility concerns is influenced by knowledge of fertility decline. Similarly, the association between higher education and absence of pregnancy timing consideration corroborates previous findings; Eriksson [36] reported that highly educated women were more likely to plan to have children in the future. Hence, highly educated women might have higher health literacy, which may lead them to reflect on the timing of attempting pregnancy. Additionally, higher self-perceived pregnancy showed a similar association with the aforementioned RLP answer category. This finding may have occurred because women’s perceptions of their chances of getting pregnant may have reflected their expectations regarding this topic, in that women who have high expectations of having children considered pregnancy timing as serious and realistic.

Regarding career vision, thoughts about returning to work after delivery were associated with lower chances of opting for absence of pregnancy timing consideration. This suggests that women’s reflections on maintaining a good relationship with co-workers lead them to consider pregnancy timing intentionally and negotiate with their managers regarding pregnancy-related work conflicts [37–39].

Moreover, our results showed that lower fertility knowledge scores, absence of infertility concerns, longer intervals between the ideal age for a first pregnancy and current age, a lower number of desired children, and longer working hours were associated with the attitude favoring pregnancy delay. A previous study reported that workplace support affected women’s decision-making regarding their pregnancy timing [23]. However, our results indicated that women working ≥ 40 h had an attitude favoring pregnancy delay regardless of workplace support levels. A study on prolonged work hours showed that prolonged working hours take away one's spare time and negatively affect physical and mental health [40–42]. Therefore, we believe that women having prolonged work hours may delay their pregnancy timing due to concerns about potential difficulties in balancing childbearing and work. There were no significant results regarding workplace support and the attitude favoring pregnancy delay. A possible reason for this outcome is that workplace support, contrary to work overload, does not necessarily influence women's intentions and actions regarding becoming pregnant. A study evaluating occupational stress and related factors among Japanese working women in their reproductive years reported that stress risks related to job overload, job control, interpersonal conflict, and job suitability were associated with a high risk of psychological stress reactions [43]. It is presumed that occupational stress, such as job overload, affects the attitude favoring pregnancy delay.

Regarding reproductive vision, higher infertility concerns and a greater desired number of children were associated with lower likelihoods of belonging to both RLP categories. In contrast, higher levels of fertility knowledge were associated with a lower likelihood of attitude favoring pregnancy delay. Such knowledge encompasses not only a better understanding of women’s fertility decline but also literacy regarding possible causes of infertility and methods for prevention [11, 12]. Having this knowledge may foster “ideal behavior” regarding appropriate reflection on pregnancy timing in a woman’s life, thereby leading to enhanced RLP behavior. Therefore, instead of exclusively providing information on fertility decline and infertility risk, education that addresses specific actions effective for safe and healthy pregnancies could empower Japanese women to avoid behaviors related to delayed pregnancy. Although high self-perceived pregnancy probability reduced the risk of neglecting pregnancy timing, it was not associated with reduced attitudes favoring pregnancy delay. A possible reason for this could be that high self-expectation regarding pregnancy may lead women to feel they still have time, preventing them from taking immediate action to conceive.

Conversely, no career vision factor was associated with both RLP answer categories. Nonetheless, thinking about returning to work after delivery was associated with reduced chances of a participant being included in the absence of pregnancy timing consideration category, whereas long working hours were associated with higher attitudes favoring pregnancy delay. These differing results regarding different career vision factors imply that the two corresponding topics (pregnancy timing consideration and attitude favoring pregnancy delay) vary for women. That is, although women who think about returning to work after delivery may consider their pregnancy timing, such consideration may not lead to actual pregnancy-related behaviors. Conversely, women’s current employment status, which has been shown to directly affect women’s physical and psychological conditions, may lead to actual pregnancy-related behavior. Therefore, maintaining Japanese women’s good physical and psychological condition through proper management of their employment status (e.g., an appropriate number of working hours) could be helpful in minimizing delayed pregnancy timing. Ever since the enforcement of Equal Employment Act 30 years ago, women's careers have developed in Japan [44], and they are expected to work longer to compete with men professionally. Nonetheless, we concur with a previous study of the Japanese work system aimed at promoting a balance between people’s professional and personal lives [45]; additionally, in our opinion, this new work system should focus on promoting childbearing in Japan and on providing pre-pregnancy support for women to prevent delayed pregnancy. Our data indicate that lower fertility knowledge and burdensome work conditions are associated with attitudes favoring pregnancy delay, which directly supports educational interventions to improve fertility literacy and labor policies that reduce excessive working hours.

International findings showing that low fertility knowledge and intensive work conditions contribute to delayed childbearing align with our results. Reportedly, insufficient awareness of age-related fertility decline reduces timely pregnancy planning [11, 46, 47], while long working hours and inflexible schedules discourage pregnancy intentions in various low-fertility contexts [48–50]. Evidence from Singapore further indicates that flexible work arrangements can increase fertility intentions among women [51]. Additionally, comparative demographic research shows that delayed first births significantly contribute to reduced completed fertility in many high-income countries [52–54].

Several alternative explanations should be considered when interpreting these findings. Women’s infertility concerns and perceived pregnancy probability may be influenced not only by biomedical knowledge but also by media narratives, cultural expectations around age and fertility, and social comparisons via online platforms, which were not captured in our dataset. Furthermore, the association between long working hours and attitudes favoring pregnancy delay may partly reflect workplace norms, such as expectations to prioritize work over family or anticipated stigma related to pregnancy, rather than work hours alone. Potential biases also warrant caution. These factors, combined with Japan’s broader societal context of long working hours and persistent gendered expectations regarding childcare, may further contribute to delayed childbearing.

While the dataset was collected in 2017, this period captures essential structural and psychosocial conditions that continue to shape reproductive decision-making among women in their 30s. These mechanisms have continued into the 2020 s, supporting the applicability of our findings in the present context.

### Limitations

Though this study has significant contributions, it also had some limitations. First, it had a measurement limitation regarding the “behavior” variable. We did not evaluate any specific behaviors related to participants’ avoidance of delayed pregnancy timing, mainly because the related actions might have varied widely. Therefore, we measured the attitude favoring pregnancy delay using a question item instead. Future studies are required to clarify specific actions that relate to fertility behaviors among married women who wish to have children and determine which factors of reproductive and career visions affect such behaviors. Moreover, the measures of concepts such as pregnancy timing consideration and attitude favoring pregnancy delay were based on a single item assessments, which may limit the construct validity of this variable. However, the response categories allowed for meaningful differentiation among participants, and the observed associations aligned with the theoretical framework. Future studies should incorporate multi-item scales to capture this construct more comprehensively. Regarding our statistical results, due to issues with the distribution of sample sizes, results such as self-perceived pregnancy probability should be interpreted with caution.

Second, the survey was conducted online; hence, selection bias toward women who were already interested in fertility and women who had tablets or smartphones (needed to access the online survey and might be accustomed to using them) cannot be ruled out. Notably, about half of the study participants had a university degree or higher, while demographically, 36% Japanese women aged 25–35 years have a university degree or higher [55]. This data might also explain the selection bias. Despite this bias, since the results were analyzed for reproductive vision and career vision aspects, there is a need to develop support systems that promote reproductive-aged women’s RLP, regardless of education level or employment status. Moreover, owing to these characteristics, our results could also have been affected by high levels of health literacy within the sample; therefore, future studies exploring the relationships between aspects of RLP and fertility knowledge are warranted.

## Conclusion

This study revealed that participants’ reproductive and career visions were associated with both RLP answer categories: *absence of pregnancy timing consideration* and an *attitude favoring pregnancy delay*. Specifically, infertility concern, as a component of reproductive vision, were associated with both RLP answer categories. Other reproductive and career vision aspects associated with RLP differed by group, and considering a return to work after delivery was a preventive factor for the absence of pregnancy timing consideration. Finally, higher comprehensive fertility knowledge and working shorter hours were preventive factors for an attitude favoring pregnancy delay.

## Supplementary Information


Supplementary Material 1.


## Data Availability

The datasets used and analyzed during the current study are available from the corresponding author on reasonable request.

## Abbreviations

AOR: Adjusted odds ratio
CI: Confidence interval
COR: Crude odds ratio
OR: Odds ratio
RLP: Reproductive Life Plan
STIs: Sexually transmitted infections
