# Correction: Factors of the reproductive life plan for married and employed Japanese women considering having children

**DOI:** 10.1186/s12905-026-04830-3

**Published:** 2026-08-28

**Authors:** Shizuko Angerhofer, Mikako Yoshida, Fumi Atogami, Yasuka Nakamura, Yoko Takeishi, Toyoko Yoshizawa

**Affiliations:** 1 Faculty of Nursing, Iwate Prefectural University, 152-52, Sugo, Takizawa, Iwate 020-0693 Japan; 2 Department of Women’s Health & Midwifery, Tohoku University Graduate School of Medicine, Sendai, Miyagi Japan; 3 Faculty of Life Sciences, Kumamoto University, Kumamoto, Kumamoto Japan; 4 Department of Nursing, Yamagata Prefectural University of Health Sciences, Yamagata, Yamagata Japan; 5 Mie Prefectural College of Nursing, Tsu, Mie Japan

**Correction: BMC Women's Health 26, 414 (2026)**

**https://doi.org/10.1186/s12905-026-04600-1**

In article [1], the author has identified that Fig. 1 is displayed incorrectly in the PDF version.

The incorrect and correct images are displayed below.

The original article has been corrected.

Incorrect Fig. 1:

**Fig. 1:**
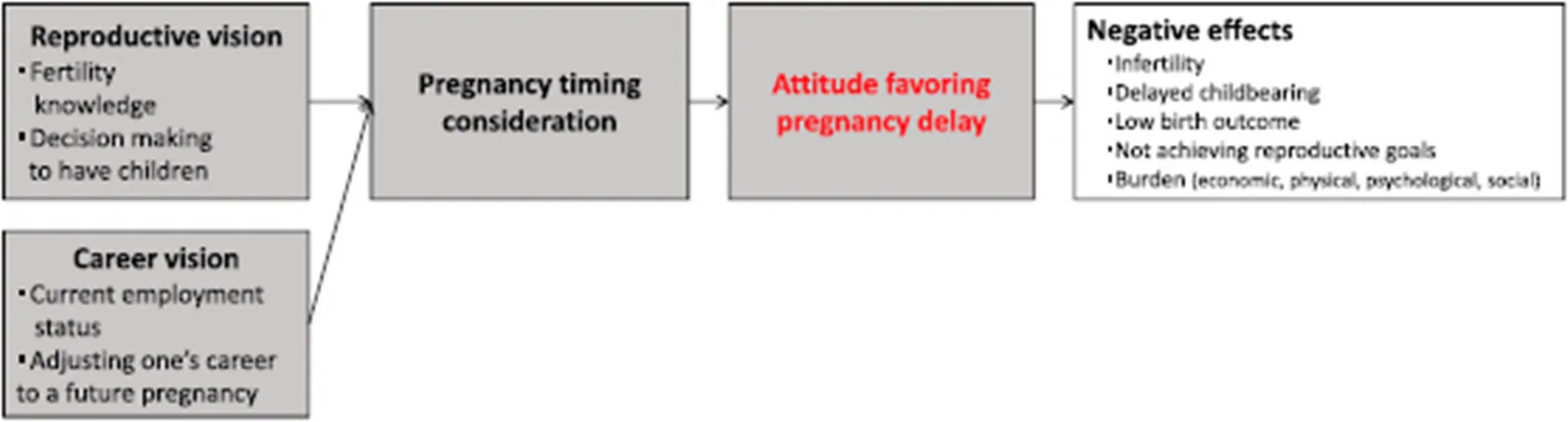
Conceptual framework

Correct Fig. 1:

**Fig. 1:**
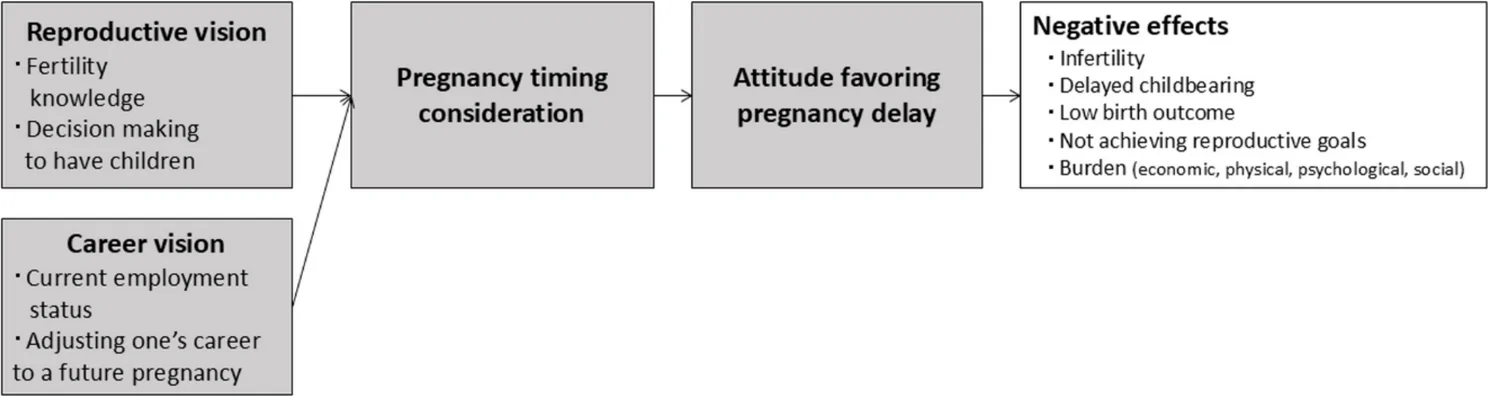
Conceptual framework

## References

[1] Angerhofer S, Yoshida M, Atogami F, et al. Factors of the reproductive life plan for married and employed Japanese women considering having children. BMC Womens Health. 2026;26:414. 42286637 10.1186/s12905-026-04600-1 PMC13487961

