# Supplementary material for: Factors of the reproductive life plan for married and employed Japanese women considering having children
Source: BMC Womens Health. 2026 Jun 13;26:414. doi: 10.1186/s12905-026-04600-1 (PMC13487961; doi:10.1186/s12905-026-04600-1)
Supplement: Supplementary file 1 [file 12905_2026_4600_MOESM1_ESM.docx]

Questionnaire of RLP with reproductive and career vision (29 items)

<Inclusion Criteria>

i. Japanese female aged 30 to 39 years old
ii. Employed
iii. Married
iv. Having no children
v. Wishing to have children in the future
vi. Not pregnant at present

<Exclusion criteria>

i. Having performed hysterectomy
ii. Unable to understand Japanese

1. Please answer the following questions.

| 1-1 | How old are you? | y/o |
| --- | --- | --- |
| 1-2 | Which of the following best describes your highest level of education? | 1. Junior-High School  2. High School  3. Vocational School  4. Junior College  5. University: Undergraduate  6. University: Graduate |
| 1-3 | How old is your husband? | y/o |
| 1-4 | How many years have you and your husband been married? | Year(s) |
| 1-5 | Would you be okay if you were to get pregnant right now? | 1. Right now would be okay  2. Not now owing to work-related reasons  3. Not now owing to non-work-related reasons.  4. Never considered |

2. Please answer the following questions based on your current thoughts regarding having children.

| 2-1 | How many children do you want to have? |  |
| --- | --- | --- |
| 2-2 | At what age would you like to have your first child? | y/o |
| 2-3 | At what age would you like to have your last child? | y/o |
| 2-4 | What would be the chance, in percentage, for you to get pregnant? | ％ |
| 2-5 | What would be the maximum age that you deem would still allow for you to naturally have children? | y/o |
| 2-6 | Have you ever been concerned about having infertility issues? | 1. No  2. Yes |

3. Please select the number that best reflects your opinion regarding the following statement.

| 3-1 | If a couple have been trying to conceive without using contraception for over a year and have not become pregnant, they are considered infertile. [True] | 1. True  　2. False  　3. Don’t know |
| --- | --- | --- |
| 3-2 | One in six couples suffers from infertility. [True] | 1. True  　2. False  　3. Don’t know |
| 3-3 | When a couple cannot have children, it is usually due to female factors. [False] | 1. True  　2. False  　3. Don’t know |
| 3-4 | Women's fertility decreases after the age of 36. [True] | 1. True  　2. False  　3. Don’t know |
| 3-5 | Even women who have never had a menstrual period can become pregnant. [False] | 1. True  　2. False  　3. Don’t know |
| 3-6 | Currently, women in their 40s are just as likely to become pregnant as women in their 30s. [False] | 1. True  　2. False  　3. Don’t know |
| 3-7 | Women who are more than 13 kg overweight are less likely to become pregnant. [True] | 1. True  　2. False  　3. Don’t know |
| 3-8 | Smoking decreases female fertility, but does decrease male fertility. [False] | 1. True  　2. False  　3. Don’t know |
| 3-9 | Men are fertile if they have sperm. [False] | 1. True  　2. False  　3. Don’t know |
| 3-10 | If a man can get an erection, he can get someone pregnant. [False] | 1. True  　2. False  　3. Don’t know |
| 3-11 | Men who contract mumps after puberty are more likely to become infertile. [True] | 1. True  　2. False  　3. Don’t know |
| 3-12 | Those who have had a sexually transmitted infection are more likely to have difficulty conceiving. [True] | 1. True  　2. False  　3. Don’t know |
| 3-13 | Having a healthy lifestyle affects your fertility. [True] | 1. True  　2. False  　3. Don’t know |

4. Please select the number that best reflects your work status and your current thoughts regarding your career.

| 4-1 | Which of the following best describes your employment status? | 1. Non-regular status with no maternity leave  2. Non-regular status with maternity leave  3. Regular status |
| --- | --- | --- |
| 4-2 | Which of the following best describes your position? | 1. Non-management  2. Management |
| 4-3 | Which of the following best describes your working hours per week? | 1. less than 40 hours  2. 40 hours or more |
| 4-4 | How would be your return to work after delivery? | 1. Quit  2. Not sure  3. Return |
| 4-5 | How much support would you expect from co-workers upon your pregnancy. | 1. None  2. Not much  3. some  4. Very much |

Angerhofer S, Atogami F, Nakamura Y, Yoshizawa T. Questionnaire of RLP with reproductive and career vision. 2017.
